# Original Remarks on the Pathological Theory, and Practical Treatment of Hydrocephalus Internus, Illustrated by Cases

**Published:** 1809-11

**Authors:** John Roberton

**Affiliations:** Author of the Practical Treatise on the Powers of Cantharides in Gleet, Leucorrhœa, and obstinate Sores, &c.; of the Treatise on Medical Police, Diet, Regimen, &c.; and Extraordinary Member of the Royal Medical Society


*Original Remarks on the Pathological Theory, and Practical Treatment of Hydrocephalus Internus, illustrated by Cases.* By JOHN ROBERTON, M. D. Author of the Practical Treatise on the Powers of Cantharides in Gleet, Leucorrhœa, and obstinate Sores, &c.; of the Treatise on Medical Police, Diet, Regimen, &c.; and Extraordinary Member of the Royal Medical Society.

THERE are, perhaps, no diseases, the nature of which are so little understood, as many which are daily to be met with among infants and children.

In the present very imperfect state of our art, even with all the advantages of the most minute detail, which those of maturer years are capable of giving of their complaints, we are often, even the most industrious among us, much perplexed and frequently bewildered in our attempts to account for various derangements in the animal economy. In children, we have not even the advantages of communicating their feelings by words, consequently, their complaints must be still a matter even of greater conjecture, than those of individuals of maturer years. Fortunately, however, in regard to the more important symptoms in the complaints of children and infants, there seems less variety than we find in those of adults; consequently, our room for conjecture in their treatment is less extensive, and our chances of success from general rules, more certain.

In these complaints, as well as in those of almost every other sort, we too often have ourselves to blame for our want of success. These errors often arise, partly, from our choosing to take for granted what had been previously said by others, without taking the trouble to think for ourselves, and partly, from our overlooking those more general affections of the system, and applying a multitude of inefficacious, if not hurtful drugs, for the removal of trifling symptoms, which, comparatively considered, are of no importance, while the more important symptoms are permitted to commit the most dreadful ravages on the system, which must ultimately, if unchecked, destroy the existence of the patient.

This reasoning may be applied to, perhaps, almost every disease which Nosologists have, by torturing and twisting, at last been able, in order to suit some particular theory, to form them into what they call a system. But, on the present sent occasion, I would more particularly apply such reasoning to Hydrocephalus Internus. I am of opinion, now that small-pox has been driven out of this country, that this is *the disease* of children; that as many, if not more, die of it than ever died of the small-pox; but the names we have been in the habit of applying to this affection, such as *spaining brash, bowel-hive, milk-fever, draughts, convulsions, &c.* while they yielded no information to the scientific inquirer, only served to bewilder the generality of people respecting its real nature. It will, however, be at all times found, that upwards of ninety cases in the hundred who die of either what is known among nurses, old women, and old women practitioners of physic, of which there are not a few, by the above and some other as absurd names, will be found to have water in the ventricles of the brain on opening the head after death. Now, as effusion of this nature can only take place, either in consequence of previous inflammatory action, or from a long continuance of debility from disease, this effusion must be caused by one or other of these affections. Certainly then, the medical attendant, if he knows any thing at all beyond his *bow*, and too often his ridiculously affected *grimace*, must be able to ascertain to which of these states the system is most liable during the prevalence of certain symptoms, and previous to the termination of this disease by effusion. This is, at all times, easily known by the state of the pulse, and by the general habit of body of the patient.

In by far the greater number, a generally inflammatory state of the system prevails previous to effusion into the ventricles; and certainly, in this form of the disease, it is much more manageable than in the other, where every individual function has become debilitated—often beyond the power of the most active remedies to restore to any permanent degree of healthy action.

The degree of torpor of certain parts of the system, as will appear from some of the cases which I shall subjoin, is, even while the system in general is, so far as we can judge by the pulse, &c. in the highest state of inflammatory action, astonishingly great. I may observe, that I have seen this state of the system become much worse a longer or shorter time before death, according to circumstances. I have even seen the body become so torpid, that neither warm baths would warm it, nor would any sort of blisters materially affect the skin. As this, however, is almost never present in the commencement of that that diseased action, which if unchecked almost always terminates in effusion into the ventricles, we have ourselves to blame if ever we allow it to supervene, which, while it requires the most active treatment, may in almost every instance be prevented by it.

I may also subjoin one remark, previous to my detail of the cases, which I shall do in confirmation of what I have adduced, that had I, as a single individual, treated complaints of many children, whose cases now recur to my recollection, both under my own immediate care, and also under the care of those whose practice I have had opportunities of witnessing, they might have been entirely relieved of their diseases, but which, from that want of energy, which we too often have occasion to witness in the practice of physic, terminated fatally.

In this place I shall decline making any further remarks on the nature and treatment of this very generally prevailing disease, and refer my readers to my Treatise on *Medical Police*, now published, where a concise, but I hope, satisfactory examination of its nature, treatment, &c. are inserted.

Case I. On the 19th of June, 1806, a boy, aged 4 ½ years, was seized with stupor and almost constant inclination to sleep, during which he breathed with great difficulty, and his pulse beat with much rapidity and strength. A blister was immediately applied over his head, and, next day, it was found necessary to renew it, as the first produced only very partial vesication. Calomel and jalap, in large doses, mean while, produced only slight catharsis. The second blister did much better than the first, and, on the 22d, he seemed quite relieved; could answer any question that was put to him, and even was out of bed an hour or two. I ordered the cathartic powders to be continued.

On the 23d, his eyes were very much inflamed, and he complained of great pain in his forehead. In the evening he was seized with phrenitis, and next day died in convulsions.

On opening the cranium, the meningeal vessels were very distinct; the blood vessels from the corona backwards, extraordinarily turgid; and adhesions between the opposite hemispheres of the cerebrum, uncommonly strong. The vessels in the ventricles were extremely turgid, especially those of the choroid plexus, and there was a considerable quantity of watery fluid in all the cavities of the brain.

Case

Case II. Miss ———, aged 8 years, was in June, 1805, affected with pain of head, particularly when she stooped, and her eyes were of a dull, heavy appearance. Her bowels were torpid, and her pulse 160 and strong; her countenance was pale, shrunk, one of her eyes squinted a little; and although she had complained only two days, she was considerably emaciated. A cap blister was applied over her head, and smart cathartics of calomel and jalap immediately administered. After the blister had remained twelve hours, it produced no other effect than that of slightly inflaming the part to which it had been applied. I now sprinkled powdered cantharides over the whole surface, formerly occupied by the blister, which, by next day, produced several vesicles. During the last twenty-four hours, she had taken grs. xvi of calomel, and the same quantity of jalap, which brought on only slight catharsis. The stools were very black and fœtid, but there was no alleviation of her complaints. I accordingly ordered the calomel powders to be continued till her stools assumed a more healthy appearance; the blistered part to be kept open with epispastic ointment; and leeches to be applied to the temples. Together with the other applications, the leeches were applied thrice in ten days, when her pulse sunk to 100 per minute, her eyes became more lively, stools became of a natural colour, and although, during this time, she had taken grs. vi of calomel and the same quantity of jalap daily, she was much stronger than before the exhibition of these medicines. I therefore ordered the blister to be kept open for some days and the bowels to be freely opened. She is now (1809) free from every complaint, having had no relapse since the above period.

Case III. A stout boy, aged 5 years, had never had any complaint till within two days before I visited him, when he became drowsy, did not seem to sleep, and complained of severe pains in his head. When I saw him, his pulse was 190 per minute, his face pale, pupils of his eyes dilated; and, while he cried, he rolled them so as sometimes to turn the pupils completely under the eyelids. His bowels were regular, but the stools were very fœtid. I ordered a cap blister to be applied, and smart cathartics to be taken. The blister was applied twice before a complete vesication was formed; the cathartics operated well; and in two days his stools became much less fœtid than formerly, during which time he voided no worms. In two days more, the pupils were contracted, and he was out of bed and amusing himself.

himself. I, however, ordered the cathartics to be continued during a few days, and the blister to be kept open. This patient in 1809 had no relapse.

A fortnight after, this boy's sister, 18 months old, became what the parents termed feverish, which had continued a week before they sent for assistance. When I saw her, her mother informed me that she was much relieved to what she had been, and had slept several hours during the day. The child was much emaciated; pulse 90. Two hours after, she died in convulsions; and on opening the cranium, I found the ventricles filled with water.

Case IV. A boy, aged 3 years, was affected with pains in his bowels; he was costive, his pulse was 160 per minute, very strong, and he screamed almost incessantly.

I ordered him grs. xxiv of submuriate of mercury, divided into xii powders, one to be taken every two hours till they operated briskly, and the warm bath to be used. When he had taken eight of these powders, he had had no loose stool. I immediately odered an injection, and a cap blister, and desired his mother to continue giving him the powders. The pulse was then 190. By the time he had taken twelve powders, he had four or five fœtid green stools. The extremities now becoming cold, he was put in the warm bath, cathartics and injections were continued, and in a week he was completely well, his pulse having fallen to 100 per minute. The parents of this patient soon after left Edinburgh, consequently I do not know whether the child continued free from disease.

Case V. A female child, aged 8 months, had, for three months past, been affected with cough, had frequently passed numbers of ascarides by stool, for the removal of which she had taken vermifuge medicines; in other respects, she was perfectly well. Her pulse suddenly rose to 180 per minute, when, suspecting her teeth to be the cause of this symptom, her gums were freely scarified, a cap blister was ordered, and smart cathartics were prescribed. Owing to the timidity of the mother, the blister was not applied till next day. The cathartics operated well, but she voided no ascarides. The child's senses remaining perfectly entire, the pupils of the eyes contracted as in health, and being without any other sign of disease, except the high pulse, she suddenly became convulsed, and her eyes turned upwards and fixed; the gums were again freely scarified: beef tea injections were given, and the warm warm bath used, which seemed to relieve her for a few minutes, but she relapsed and died in two hours.

On dissection, the adhesions between the dura mater and cranium were uncommonly strong; indeed, so much so, that I found it impossible to separate them without doing considerable injury both to the skull and the brain. The blood-vessels, which were not ruptured, were greatly distended, and water seemed to flow from almost every part of the substance of the brain. Independently of the quantity of fluid thus discharged, on removing the brain from the cranium there were several ounces collected at the base of the skull.

In this case I had an opportunity of opening the thorax and abdomen, but no collection of fluid was found in them, nor did they seem at all in a diseased state.

Case VI. A female child, aged 14 months, became restless, would not suck, and cried bitterly, as if suffering great pain. Her pulse was 170 and full, her bowels regular; but when cathartics were given, the stools were black, and very fœtid; next day the pulse was 180; a cap blister was applied, and, as the limbs frequently became cold, she was immersed in the warm bath to the neck. These symptoms continued a week, and during that time cathartics were freely administered, and the blister kept open; injections of beef tea, and the warm bath, were also used frequently. The pulse, however, did not decrease in frequency nor in strength till the tenth day after she was first affected, when it sunk to 170 per minute, and gradually decreased about ten beats every day till it became natural, and the child recovered completely. About two years after this, the child was again similarly affected; but as the mother would not allow such active measures to be used, death of course was the consequence, and on opening the cranium the ventricles were found filled with fluid.

Case VII. A stout boy, aged 5 years, was suddenly affected with pains in his bowels and head; the pulse 180, and very strong. His bowels were ordered to be opened with calomel and jalap; leeches to be applied to the temples, and a cap blister to the head. His stools were very dark coloured, but there were no worms observed in them; in 24 hours he took half a drachm of submuriate of mercury, and the same of jalap, and his bowels were only moderately opened. I also prescribed beef tea injections, to to defend as much as possible the intestines against the action of such immense doses of physic. The mother, however, would not consent to put the blister on his head, neither were the leeches applied. Two days after, the pulse beat 250 per minute, and every day the progress of emaciation was more and more observable. The parents, therefore, became alarmed, and at last consented to apply the blister; but before vesication was produced, it was necessary to apply this twice, and the strongest epispastic ointment was barely sufficient to keep the sore open. In two days the pulse was 200; the child's senses were perfectly entire; the eyes quite natural; yet still it was necessary to give strong doses of physic to keep the bowels open. The face became œdematous, so that the eyes were scarcely to be seen for two or three days; the pulse being still 190, and very strong. The parents now became obstinate, and insisted on the blister being healed, which was accordingly done, notwithstanding all I could say to the contrary. For three weeks the pulse was never below 180, and it was frequently above 200 in a minute. Yet, the child was perfectly sensible till within half an hour of his death, when he became insensible, and died in convulsions; the pupils, however, never being dilated. The parents would not allow the head to be opened.

Case VIII. A female child, aged one year, had been restless and unwell for a whole day; her pulse beat 180 per minute, and skin natural. Two of the incisores of the under jaw had completely cut the gum, but no other teeth seemed to be near the cutting. The child had been constantly costive since she was born, and had frequently been troubled with a slight cough. After having been repeatedly informed, that, till the morning of the day on which I was first desired to visit her, she had been in her usual health; I was at length told by the nurse, that three or four days before, the child's eyes appeared wild, and that she turned them as if looking towards her nose; the same day she also stretched her arms, fingers, and legs, as if in fits, and screamed dreadfully; but as these symptoms soon abated, she (the nurse) did not wish to alarm her mother by a relation of them. I prescribed calomel powders, in smart doses, and a cap blister to be applied over the head. The blister rose well, but the bowels were no more than moderately opened, even after gr. xviii. of calomel had been given, and the stools were of a dark brown colour. On the following day an erysipelatous affection was perceived on the fore part of the left shoulder; a ghastly a ghastly paleness, or rather blueness, was observable in the face, particularly under the eyes and round the mouth, and the features were remarkably shrunk. Her pulse being 180 and strong, the powders were ordered to be continued in sufficient doses, to keep the bowels regular, beef tea injections to be given, and the warm bath to be used. These produced no alleviation of the symptoms, and the screaming and squinting of the eyes again returned. I now ordered pledgits of cloth, dipped in alcohol, to be applied to the inflamed part, which gave instant relief; and now, as it began to decrease above, it appeared inflamed further down the arm, but was finally cured by the application of the alcohol.

Three days after the first attack a slight convulsive affection was observed, though it soon went off. The blister was therefore to be kept open, the calomel powders to be continued, and the warm bath to be used.

On the fifth day the pulse was 156, and not so strong as formerly.

On the eleventh day, no unfavourable symptoms had occurred for a week before, and I ordered that strict attention be paid to the bowels. The blister had been healed two days.

In September 1809, this child remained perfectly well.

Case IX. A female child, aged 8 months, uncommonly stout, and till now, never seeming to complain; the pulse being 190, and very strong, was, four days previous to the time I was desired to visit her, affected alternately with drowsiness and severe screaming. The bowels were constipated, and as several of the teeth seemed nearly thro' the gums, they were cut upon. A cap blister and smart cathartics were also prescribed; and during next day, the pulse was only 100 per minute. While I was almost regretting that such active treatment had been used, when perhaps much more mild treatment might have answered the purpose, the symptoms were in a few hours aggravated, the pulse being 180, but much weaker than formerly. There was considerable difficulty in voiding urine, and the child had something like convulsions, though they were very slight. The extremities now became cold, on which account I prescribed the warm bath to be used frequently, and the same evening, while in the cradle, after using the bath, she died. Her parents did not observe whether she died in convulsions, and they would not allow the head to be opened.

Case X. A boy, eight months old, was, six months ago, affected with difficulty of breathing, accompanied with a cough somewhat resembling croup. A blister was then applied to the breast, with the administration of cathartics, which relieved these symptoms; but, on the 1st of January, he was again affected with the same symptoms as formerly, according to his mother's account; for I did not see him till the 9th of January. He had then several teeth; his pulse intermitted; his face was alternately red and pale; the pupils of his eyes were contracted as if in health; but there was a certain dullness in them, which, however, his mother asserted was their natural state, as she never observed them otherwise. I scarified the gums freely, and ordered smart cathartics, and in the evening found that several teeth had appeared in consequence of scarifying them, but without any alleviation of his other complaints. I therefore ordered a cap blister to be applied; and next morning, I was informed it had produced no effect on the head. I called an hour after, and was told that my patient had died a few minutes before.

Dissection, Jan. 10. The pupils of the eyes were so contracted as only to be about a quarter of an inch in diameter; and the tongue firmly impacted between the jaws. On cutting through the integuments of the head, a considerable quantity of blood flowed; water and blood flowed while sawing through the cranium; and then watery blood issued from the diploe, about three ounces in quantity. From the vessels of the dura mater, after the skull cap was removed, several ounces of blood flowed, and all the vessels of the membranes were uncommonly turgid, particularly those ramifying on the pia mater. On cutting into the substance of the brain, blood flowed; the ventricles were full of water; the choroid plexus greatly distended, resembling hydatids, and parts of it were uncommonly red. Coagulated blood existed on the surface of the cerebellum, where it comes in contact with the cerebrum, and the cavities in the base of the cranium were filled with water tinged with blood.

Case XI. January 10. A female child, aged 3 years, was, about a week before I visited her, exposed to the effects of cold damp air, in common with two other children of the same family. They were all seized with a catarrhal affection, though the other two were now nearly recovered. Her parents, of their own accord, had given her, a few days before before, a dose of physic which operated freely. The stools were of a brown colour. When I saw her, for the first time, she seemed restless; her pulse was 160, of ordinary strength; her eyes lively; but she had had no passage in her bowels since she took the cathartic.

I prescribed submuriat. hydrarg. grs. xxx. Sacc. alb. q. s. Divide in vi. æq. One to be taken in jelly every four hours, till they operate freely; and a beef tea injection to be given immediately.

On the 21st of January, by ten o'clock, sixteen hours after the physic was prescribed, she had taken four of the above powders, yet no effect was produced on her bowels, but beef tea injections were followed by a plentiful discharge of black fæces.

Her eyes looked very heavy, yet she was quite sensible; the pupils dilated when the light was obstructed, but on the application of light to them, they did not contract sufficiently. I ordered the powders to be continued, the beef tea injection to be repeated, the warm bath to be used, and a cap blister to be applied. In the evening the powders were all taken, but they had produced no evacuation, and the beef tea injections brought nothing away with them; the warm bath relieved her at first, but now it fatigued her much. I accordingly ordered an emetic to be given, and friction to be used all over her belly. As her mouth had become very sore, I ordered the calomel powders to be omitted, and after the vomit had operated, to give an injection of a decoction of senna; a tea spoonful of the decoction to be swallowed every half hour, till evacuation of the bowels was procured, and the friction to be persevered in.

On the 22d, the emetic had operated well, but still no evacuation of her bowels had occurred, although she had taken since the operation of the vomit about three ounces of a very strong decoction of senna. The pulse was now 180, and her abdomen was so sore to the touch that she could not allow friction to be applied. I therefore prescribed Ol. Ricini ℥ß. to be taken immediately. About two hours after its administration, she had a small evacuation, very fœtid, hard, and black, and was relieved for a few minutes, when she became drowsy, and her extremities cold. The warm bath was applied, and orders left that another calomel powder should be given in a few hours, if she had no evacuation.

On the 23d, the powder given at nine o'clock produced two plentiful stools before morning, not so hard, but as black and fœtid as the former. I ordered the powders to be repeated, and a beef tea injection to be given. She seemed much relieved in consequence of it.

On the 24th, one powder was ordered to be given, and the warm bath and injection to be repeated; the pulse being 130.

On the 25th, she had several black stools since the preceding day, which were thin in consistence, but still very fœtid. I ordered ℥ß. of Ol. Ricini to be taken this night at bed-time.

On the 26th, she had free evacuation of her bowels since the previous day; the last stool which she had being natural in colour, I desired that the Castor oil should be continued, or a decoction of senna, to procure one or two stools every day, and the blister on her head to be kept open. The pulse was now 120, and not very strong.

On the 28th, she had stools natural in colour and regular, and had ate a piece of chicken with a keen appetite; her pulse being 110, I ordered the blister to be healed, and occasional doses of physic to be taken.

The child continued well when the family went into the country about a year and a half afterward.

*Princes Street.*

